# Hearing Rehabilitation With a Chat-Based Mobile Auditory Training Program in Experienced Hearing Aid Users: Prospective Randomized Controlled Study

**DOI:** 10.2196/50292

**Published:** 2024-02-09

**Authors:** Jae Sang Han, Ji Hyung Lim, Yeonji Kim, Aynur Aliyeva, Jae-Hyun Seo, Jaehyuk Lee, Shi Nae Park

**Affiliations:** 1 Department of Otorhinolaryngology–Head and Neck Surgery, Seoul St. Mary’s Hospital, College of Medicine, The Catholic University of Korea, Seoul, Republic of Korea; 2 Department of Pediatric Otolaryngology, Cincinnati Children’s Hospital, Cincinnati, OH, United States; 3 Nara Information Co, Ltd, Seoul, Republic of Korea

**Keywords:** hearing loss, hearing aids, hearing rehabilitation, auditory training, mobile program

## Abstract

**Background:**

Hearing rehabilitation with auditory training (AT) is necessary to improve speech perception ability in patients with hearing loss. However, face-to-face AT has not been widely implemented due to its high cost and personnel requirements. Therefore, there is a need for the development of a patient-friendly, mobile-based AT program.

**Objective:**

In this study, we evaluated the effectiveness of hearing rehabilitation with our chat-based mobile AT (CMAT) program for speech perception performance among experienced hearing aid (HA) users.

**Methods:**

A total of 42 adult patients with hearing loss who had worn bilateral HAs for more than 3 months were enrolled and randomly allocated to the AT or control group. In the AT group, CMAT was performed for 30 minutes a day for 2 months, while no intervention was provided in the control group. During the study, 2 patients from the AT group and 1 patient from the control group dropped out. At 0-, 1- and 2-month visits, results of hearing tests and speech perception tests, compliance, and questionnaires were prospectively collected and compared in the 2 groups.

**Results:**

The AT group (n=19) showed better improvement in word and sentence perception tests compared to the control group (n=20; *P*=.04 and *P*=.03, respectively), while no significant difference was observed in phoneme and consonant perception tests (both *P*>.05). All participants were able to use CMAT without any difficulties, and 85% (17/20) of the AT group completed required training sessions. There were no changes in time or completion rate between the first and the second month of AT. No significant difference was observed between the 2 groups in questionnaire surveys.

**Conclusions:**

After using the CMAT program, word and sentence perception performance was significantly improved in experienced HA users. In addition, CMAT showed high compliance and adherence over the 2-month study period. Further investigations are needed to validate long-term efficacy in a larger population.

## Introduction

According to a World Health Organization report in 2021, it is predicted that a quarter of people will have some degree of hearing loss by 2050 [1]. Hearing loss is known to be related to the risk of vocational problems, depressed mood, and even cognitive impairment in adults [2-4]. Adult aural or hearing rehabilitation, a multifactorial strategy for patients with acquired hearing impairment, includes 4 main components: sensory management, instruction, perceptual training, and counseling [5]. Sensory management involves enhancing auditory function through the use of hearing aids (HAs), cochlear implants, and alternative devices. Additional perceptual training, including auditory training (AT), seems to be required because sensory management alone has limitations in improving speech perceptual ability [5,6].

Although AT is known to improve hearing performance and lower the HA return rate, it has been not sufficiently provided to patients with hearing loss due to its poor time- and cost-effectiveness [7-9]. In our previous randomized controlled trial, we reported significant improvements in consonant perception and subjective satisfaction after 8 weeks of AT consisting of in-hospital face-to-face education and at-home self-training among HA users [10]. However, we experienced the limitations of in-hospital training programs during the COVID-19 pandemic, and this led us to consider a mobile-based AT program.

Smartphone penetration is rapidly increasing worldwide, and South Korea is a leading county for smartphone use, with a penetration rate of around 93% in 2021 [11,12]. Among the older population, around 94% of those older than 60 years and 60% of those older than 70 years own smartphones, and most of them (80%) use the internet with a mobile device [12,13]. The increasing smartphone penetration rate is a global trend, and mobile-based health care programs are expected to be used effectively even in the older population [14]. We developed a new mobile AT program with a chat-based interface to increase compliance with hearing rehabilitation, with attention to the fact that most of the adult population is familiar with message apps on smartphones [15].

Several mobile and web-based AT programs have been introduced in the last decade. However, many mobile-based AT applications are simple gamified training programs for pediatric patients, and most of them have no clinical validation to support their efficacy [16]. Even though a state-of-the-art review showed AT to be effective for improving auditory perceptions [17], it is unclear whether mobile-based training will still be effective. Digital therapeutics are expected to change the future of the health care system; however, clinical validation should first be performed to prove their effectiveness [18]. In addition, the effect of AT on experienced HA users has been inconsistently reported, while the effect of AT on novel HA users is relatively clear. Of the 16 studies included in a state-of-the-art review on AT, only 3 investigated experienced HA users, and only 1 of these studies showed improvements in speech perception [19-21].

Therefore, this prospective randomized controlled study was conducted to investigate the efficacy of our novel chat-based mobile AT (CMAT) program for 3 aspects of hearing rehabilitation in experienced HA users: speech perception ability, subjective satisfaction, and training compliance.

## Methods

### Ethical Considerations

This prospective study was approved by the ethical committee of Seoul St. Mary’s Hospital (KC21EISI0525) and followed the tenets of the Declaration of Helsinki. The patient records and information were anonymized and deidentified before analysis. All participants provided written informed consent prior to commencement of the study and voluntarily participated in this clinical trial.

### Participants

A prospective randomized controlled trial with an unblinded study setting was carried out. Bilateral HA users were recruited from the department of otorhinolaryngology–head and neck surgery at a tertiary referral center between September and December 2021. Study eligibility criteria were as follows: (1) age ≥20 years; (2) bilateral moderate to severe sensorineural hearing loss (mean threshold of pure tone audiometry measured at 500, 1000, 2000, and 4000 Hz was 41 to 80 dB hearing level, and the air-bone gap was less than 15 dB hearing loss); and (3) the patient had been using bilateral HAs for more than 3 months, demonstrated sufficient functional gain, and consistently used the HAs for more than 8 hours per day. Patients were excluded if they had fluctuation in hearing loss, brain tumor, or difficulty using the program or coordinating hearing tests.

The randomization was performed using premade random cards with a 1:1 allocation ratio prepared by a contract research organization (Medical Excellence). The cards were then opened by the health care provider after obtaining informed consent. Participants allocated to the AT group (ATG) were provided instructions about the CMAT program by a coauthor (JL) and were encouraged to use it daily for 8 weeks. One CMAT session consisted of 20 questions, took about 5 to 10 minutes, and was repeated 3 times a day. Those assigned to the control group (CG) did not receive the CMAT. All participants were asked to complete audiologic tests, speech perception tests, and questionnaires before enrollment and at 1 and 2 months after the start of the study, respectively.

Assuming that the effect of offline AT and CMAT would be similar, we determined the number of target participants in the same way as in our previous study [10]. For statistical significance at a .05 confidence level with 80% power, the sample size required for the 2 groups was estimated as 18 patients per group. Allowing for a 10% dropout rate, 42 patients were estimated to be required in total.

### Chat-Based AT Program

Our CMAT program was developed in the form of a web-based program that can be accessed from computers, tablets, and smartphones. However, all participants were requested to connect with their smartphones to avoid device bias in this clinical trial. When a participant accessed a provided link, the ID and password given during participant registration were required to be entered, and a page was provided to additionally confirm the consent form for clinical trials and the collection of personal information. Afterward, a problem was presented in a chat-based interface. In the chat-based interface, we designed a conversational partner as a character representing a medical professional, intending to give users the feeling of exchanging chat bubbles with a health care provider. Participants could choose 1 of 4 answers, and a message was shown giving feedback after a problem was answered. All participants were instructed to train in a quiet place while wearing their HAs.

The rehabilitation program consisted of 2 parts: word and sentence training. For word training, a total of 1540 two-syllable Korean words that are frequently used in daily life were extracted based on data provided by the National Institute of the Korean Language. Four words with the same or similar vowels but different consonants were provided, and then one of these words was provided twice. Then, the participant was asked to choose the word that he or she heard. For sentence training, daily phrases consisting of 2- to 12-word phrases were provided, and the frequency of the words used was checked to correct or delete infrequently used words (eg, “We decided to meet at Seoul Station”). A total of 1225 sentences were composed, and a question was made for each sentence. The sentences and related questions were provided once, and 3 words similar to the correct answer were shown with it (eg, “Question: Where would you like to meet?”; “Answers: (1) Sadang Station, (2) Sung-soo Station, (3) Sung-book Station, (4) Seoul Station”).

**Figure 1.**
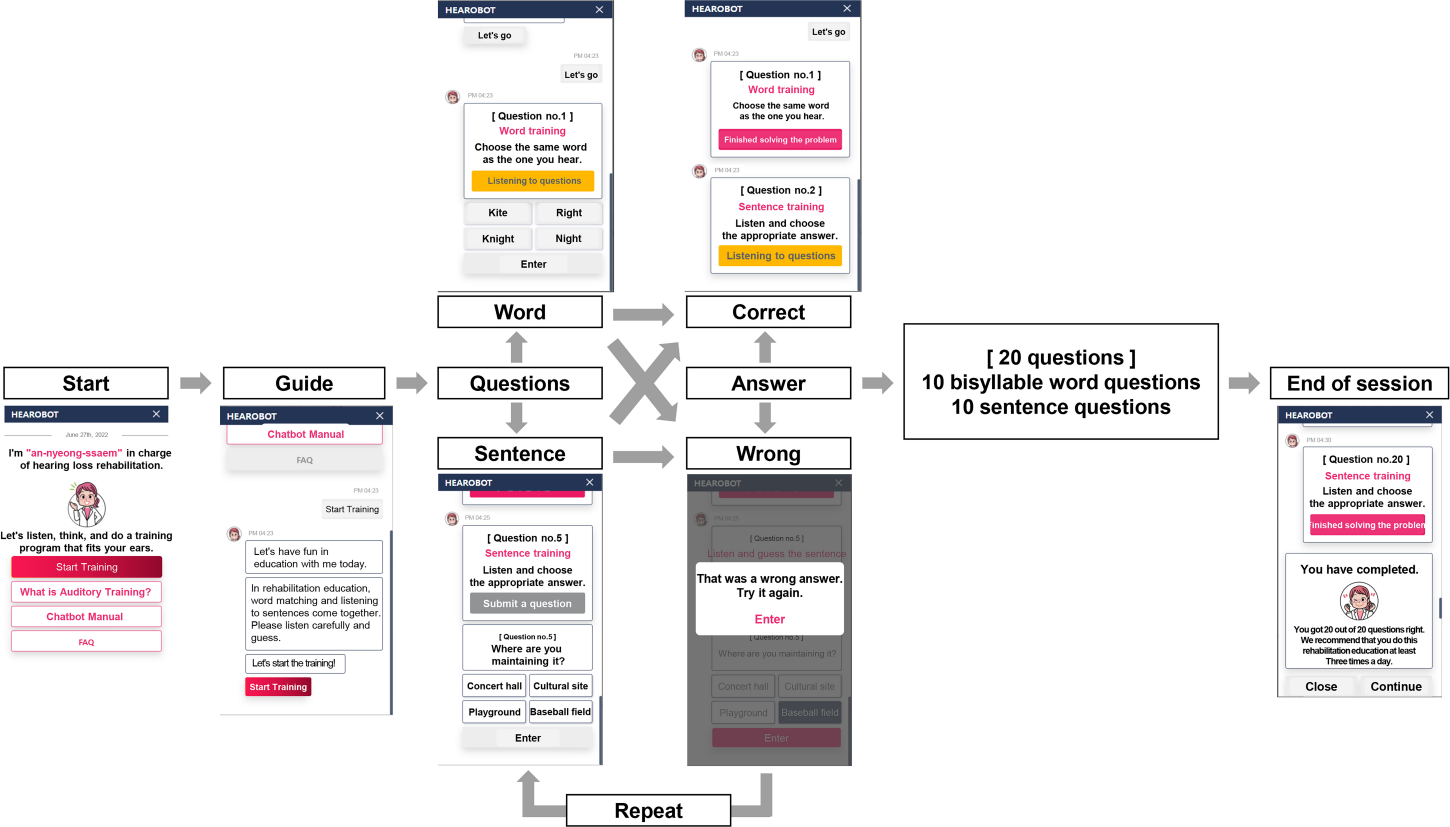
Schematic diagram of chatbot-delivered auditory training program.

This CMAT program was presented as if chatting with a virtual character. If a participant answered the question correctly, the next question among the word and sentence training question pools was shown randomly. If a participant chose a wrong answer, the question was repeated up to 3 times. Ten-word and 10-sentence questions were randomly assigned to participants for solving within a single session. AT dosage was planned to be around 30 minutes of training per day, similar to our previous study, as solving 20 questions was expected to take approximately 10 minutes. However, if a participant wanted to solve more questions, they could engage in an additional session. Participants included in the ATG were instructed to repeat this session 3 times a day, spaced out, following a similar approach to our previous study [10]. A schema of the CMAT is shown in Figure 1.

The Korean language was used in this program, and words and sentences were synthesized using Google Cloud text-to-speech software. To facilitate scalability for increases in the number of sentences or upgrades in the future, we determined that software-generated voices would be a reasonable choice. The speech was randomly generated using male or female voices provided by the software, and the default settings of the program were used for other suprasegmental characteristics of sentences, including speed, pitch, and volume. The log-in time and the number of questions solved were automatically collected by the program; however, prompts to use the CMAT were not provided.

### Outcome Measures

#### Study Design

All participants completed audiologic tests, speech perception tests, and questionnaires at the 0-, 4-, and 8-week visits, and the results were collected using Excel (Microsoft). Among these measured outcomes, speech perception test results were used as the primary end point, and questionnaire results were used as the secondary end point.

#### Audiologic Tests

Pure tone audiograms (PTAs) without HAs and sound field threshold audiometry tests with HAs were conducted to exclude changes in hearing level and inappropriate function of HAs. Speech audiometry tests, including the speech reception threshold test and speech discrimination scores, were evaluated both with and without the participants wearing HAs.

#### Speech Perception Tests

A total of 5 speech perception tests were conducted by a female audiologist in quiet conditions while the patient wore an HA. The Ling Six Sound test was conducted for phoneme detection [22], and the Vowel and Consonant Imitation Test (VCIT) was used to measure phoneme perception [23]. To evaluate word perception ability, monosyllable and bisyllable open-set tests were used [24]. Sentence perception ability was evaluated using the Korean version of Central Institute for the Deaf (K-CID) test [25].

#### Questionnaires

Subjective benefits were measured by 3 validated questionnaires. The Korean version of the Hearing Handicap Inventory for the Elderly (K-HHIE) was used to measure situational and emotional handicaps in everyday life due to hearing problems [26]. In addition, the Korean versions of the International Outcome Inventory for Hearing Aids (K-IOI-HA) and Abbreviated Profile of Hearing Aid Benefit (APHAB) tests were conducted to evaluate the subjective satisfaction with HA use [27,28].

### Statistical Analysis

Statistical analysis was conducted using SAS (version 9.4; SAS Institute). The Shapiro-Wilk test was used to examine the normality of the measured variables. Data were expressed as mean, SD, and percentage. *P* values were calculated using the chi-square test or Fisher exact test for categorical variables, the Mann-Whitney test or independent 2-tailed *t* test for pairs of independent variables, and the Wilcoxon rank sum test or paired *t* test for continuous variables. All speech perception tests were collected as percentage data, and as the data did not meet the normality assumption based on the Shapiro-Wilk test, we conducted additional analyses using arcsine transformation. Speech perception tests and questionnaire surveys were analyzed with a linear mixed-effect model and post hoc test. Random effects included the intercept, while fixed effects included group (ATG and CG), time (initial, 1 month, and 2 month), group × time interaction, and the covariate “aided 4 kHz threshold result of the right ear.” The inclusion of the aided 4 kHz threshold result of the right ear as a covariate was due to the significant differences between the 2 groups in the initial hearing test despite randomization. Further details regarding this will be provided in the *Results* section. Two covariance structures in the linear mixed model, variance components and compound symmetry, were compared. There were no substantial differences in the results of the fixed effect type 3 test, so we described the variance components estimation. The Akaike information criterion values representing model fit varied depending on the specified model, from 280 to 680. Bonferroni correction was used for multiple comparisons and adjusted *P* values are presented. Correlations between pairs of variables were analyzed with the Pearson correlation test. Differences were considered significant when the *P* value was less than .05.

## Results

### Clinical Characteristics

**Figure 2.**
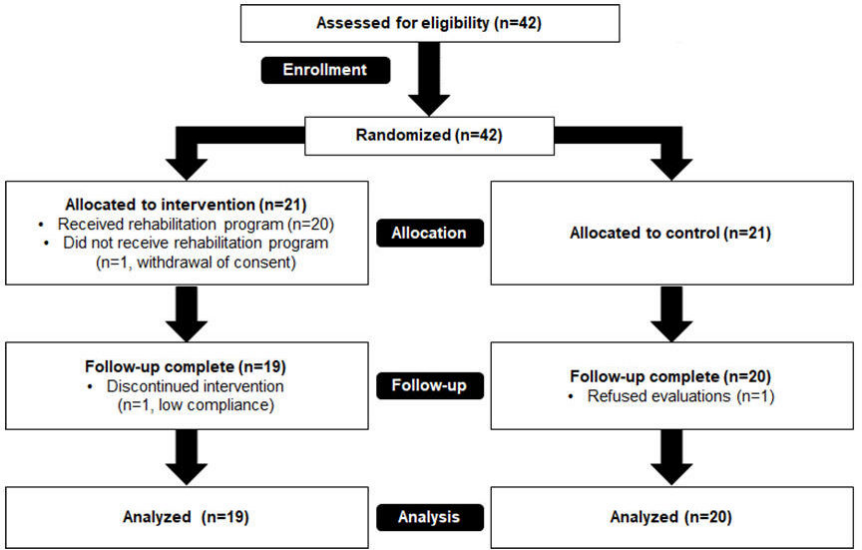
CONSORT (Consolidated Standards of Reporting Trials) flow diagram.

A total of 42 participants were initially enrolled after informed consent was obtained. The ATG and CG were each randomly allocated 21 patients, with 1 participant subsequently withdrawn from the ATG due to withdrawal of consent. During the 2-month study follow-up period, 1 participant in the ATG was excluded from the analysis due to low compliance (CMAT completion rate <50%), and 1 participant in the CG was excluded for refusing to perform speech perception tests. Therefore, 19 participants in the ATG and 20 participants in the CG were analyzed. The CONSORT (Consolidated Standards of Reporting Trials) flow diagram is shown in Figure 2. None of the participants in the ATG had any problems using CMAT during the study.

There were no significant differences between the 2 groups in age, sex, duration of hearing loss, type of HA being used, or duration of HA use. The detailed patient demographics are shown in Table 1.

**Table 1.** Clinical characteristics of the participants.

		Total (n=41)	Auditory training group (n=20)	Control group (n=21)	*P *value
Age (years), mean (SD)		70.0 (10.9)	70.5 (6.9)	69.5 (13.8)	.41^a^
**Sex, n**					.90^b^
	Male	16	8	8	
	Female	25	12	13	
**Hearing aid type, n**					.75^c^
	Complete in the canal	67	33	34	
	Invisible hearing aid	10	4	6	
	Receiver in the canal	5	3	2	
Hearing loss duration (months), mean (SD)		103.0 (86.5)	107.9 (83.3)	98.3 (91.3)	.48^a^
Hearing aid use duration (months), mean (SD)		54.5 (62.1)	56.1 (72.2)	53.0 (50.7)	.80^a^

a Mann-Whitney test.

b Fisher exact test.

c Chi-square test.

### Audiologic Evaluations

**Figure 3.**
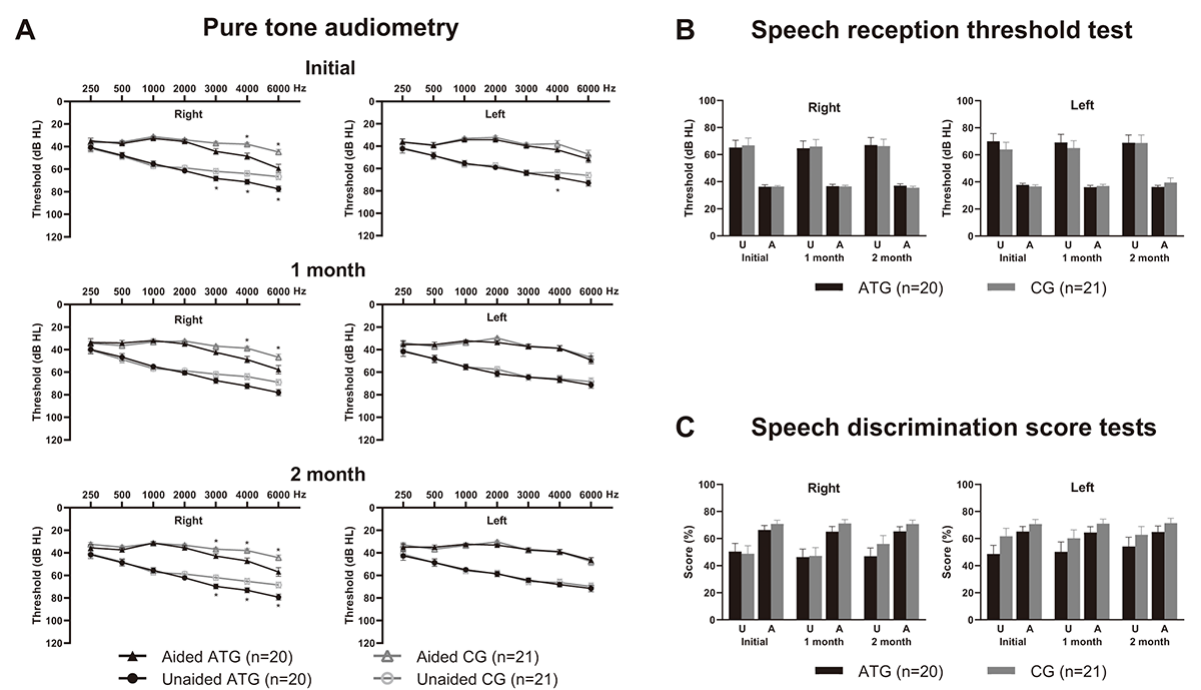
Results of audiologic tests. (A) Pure tone audiometry showing significantly higher thresholds in the auditory training group (ATG) at 3000, 4000, and 6000 Hz frequencies in the right ear. (B) Speech reception threshold test showing no significant difference between the ATG and the control group (CG). (C) Speech discrimination scores were higher in the CG without statistical significance. A: aided; dB HL: decibel hearing level; U: unaided. *P<.05; independent *t* test or Wilcoxon rank sum test.

Although we randomly allocated participants to the 2 groups, there were unintended differences in the baseline hearing tests. In the ATG, the high-frequency thresholds (3000, 4000, and 6000 Hz) of the right ear were significantly higher than in the CG in both unaided and aided conditions (independent *t* test or Mann-Whitney test, all *P*<.05). However, there was no significant difference in the left ear. In speech audiometry, mean values for speech discrimination score were higher in the CG without a statistically significant difference (independent *t* test or Mann-Whitney test, all *P*>.05). No significant changes over time were observed in the repeated audiologic tests under either unaided or aided conditions (linear mixed model, all *P*>.05). These audiologic test results are presented in Figure 3.

### Speech Perception Tests

With the exception of the vowel imitation test, the initial mean scores for the remaining 5 tests were better in the CG. Although the Ling Six Sound test, monosyllable and bisyllable open-set tests, and the K-CID test showed no significant differences (adjusted *P*>.05 for all tests; Mann-Whitney test with Bonferroni correction), the initial consonant imitation test exhibited a statistically significant difference (adjusted *P*=.003; Mann-Whitney test with Bonferroni correction). Therefore, the aided 4 kHz threshold result of the right ear, which exhibited a significant difference in the initial PTA result (*P*=.002; Mann-Whitney test), was included as a variable in the analysis of the linear mixed model, considering that high-frequency hearing loss is a well-known factor affecting hearing perception [29].

When conducting a linear mixed model that includes the 4 kHz threshold of the right ear as a variable, we observed no significant difference in the initial scores of any speech perception tests (all *P*>.05).

**Figure 4.**
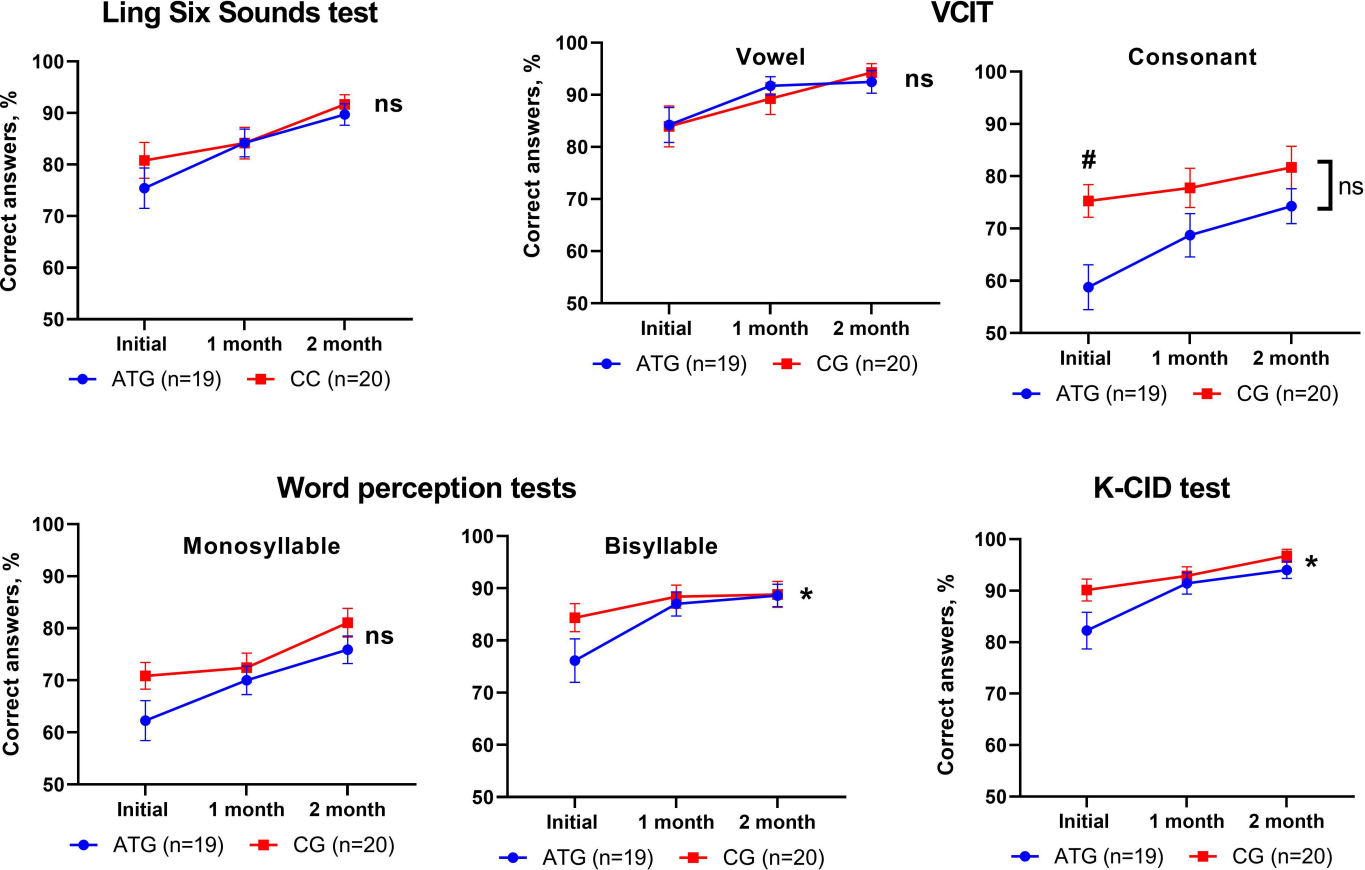
Results of speech perception tests. The auditory training group (ATG) showed significant improvement in speech perception compared to the control group (CG) in the intergroup analysis of the 2-syllable word recognition test and the K-CID (Korean version of Central Institute for the Deaf) test. ns: not significant; VCIT: Vowel and Consonant Imitation Test. *P<.05; linear mixed model.

In the Ling Six Sound test, the VCIT (vowel and consonant) and monosyllable test results showed no significant group differences (all *P*>.05, linear mixed model). However, in the bisyllable condition, the ATG demonstrated significantly better results compared to the CG (*P*=.04, linear mixed model). Similarly, in the K-CID test, the ATG showed significantly greater improvement than the CG (*P*=.03, linear mixed model). These results are visualized in Figure 4, and detailed linear mixed model analyses, including post-hoc *P* values, are presented in Table 2. Additionally, an arcsine transformation was applied to the percentage values, yielding almost the same results. Detailed values and statistical analyses of both the percentage and arcsine-transformed data are provided in Multimedia Appendix 1.

**Table 2.** Linear mixed model analysis of speech perception tests.

	Group, post hoc *P* value			Time, post hoc *P* value						Group × time, post hoc *P* value			*F* (*df*)	*P* value^a^
				Auditory training group			Control group							
	Initial	1 mo	2 mo	Initial minus 1 mo	1 mo minus 2 mo	Initial minus 2 mo	Initial minus 1 mo	1 mo minus 2 mo	Initial minus 2 mo	Initial minus 1 mo	1 mo minus 2 mo	Initial minus 2 mo		
Ling Six Sound test	>.99	.79	>.99	.05	<.001	.004	>.99	.11	.008	>.99	>.99	.03	0.64 (2, 73)	.53
VCIT^b^ (vowel)	>.99	.74	>.99	.04	>.99	.03	.21	.27	.002	>.99	>.99	.25	0.52 (2, 73)	.60
VCIT (consonant)	.20	>.99	>.99	.01	.28	<.001	>.99	.70	.15	.07	.65	.01	2.24 (2, 73)	.11
Monosyllable test	.92	>.99	>.99	.04	.17	<.001	>.99	.02	.003	.51	.35	.004	1.04 (2, 73)	.36
Bisyllable test	>.99	.85	.47	<.001	>.99	<.001	.28	>.99	.19	.30	<.001	.24	3.37 (2, 73)	.04
K-CID^c^	.83	>.99	>.99	<.001	.35	<.001	.36	.09	.001	.16	<.001	.006	3.81 (2, 73)	.03

a Group × time interaction effects compared the initial minus 1 month or initial minus 2 months.

b VCIT: Vowel and Consonant Imitation Test.

c K-CID: Korean version of Central Institute for the Deaf test (linear mixed model).

### Subjective Satisfaction

There was no significant difference between the 2 groups in all 3 types of questionnaire surveys (all *P*>.05; linear mixed model). Detailed results are described in Multimedia Appendix 2.

### Compliance With CMAT Program

Except for one participant in the ATG who withdrew consent, all participants (n=20) in the intervention group accessed the CMAT program in a mobile environment. One of 20 participants (5%) was excluded from the analysis in this study because the duration of CMAT use was less than 50% of the required AT. The remaining 19 participants (95%) completed at least 50% of the training sessions, and 17 (85%) completed more than the required AT.

**Figure 5.**
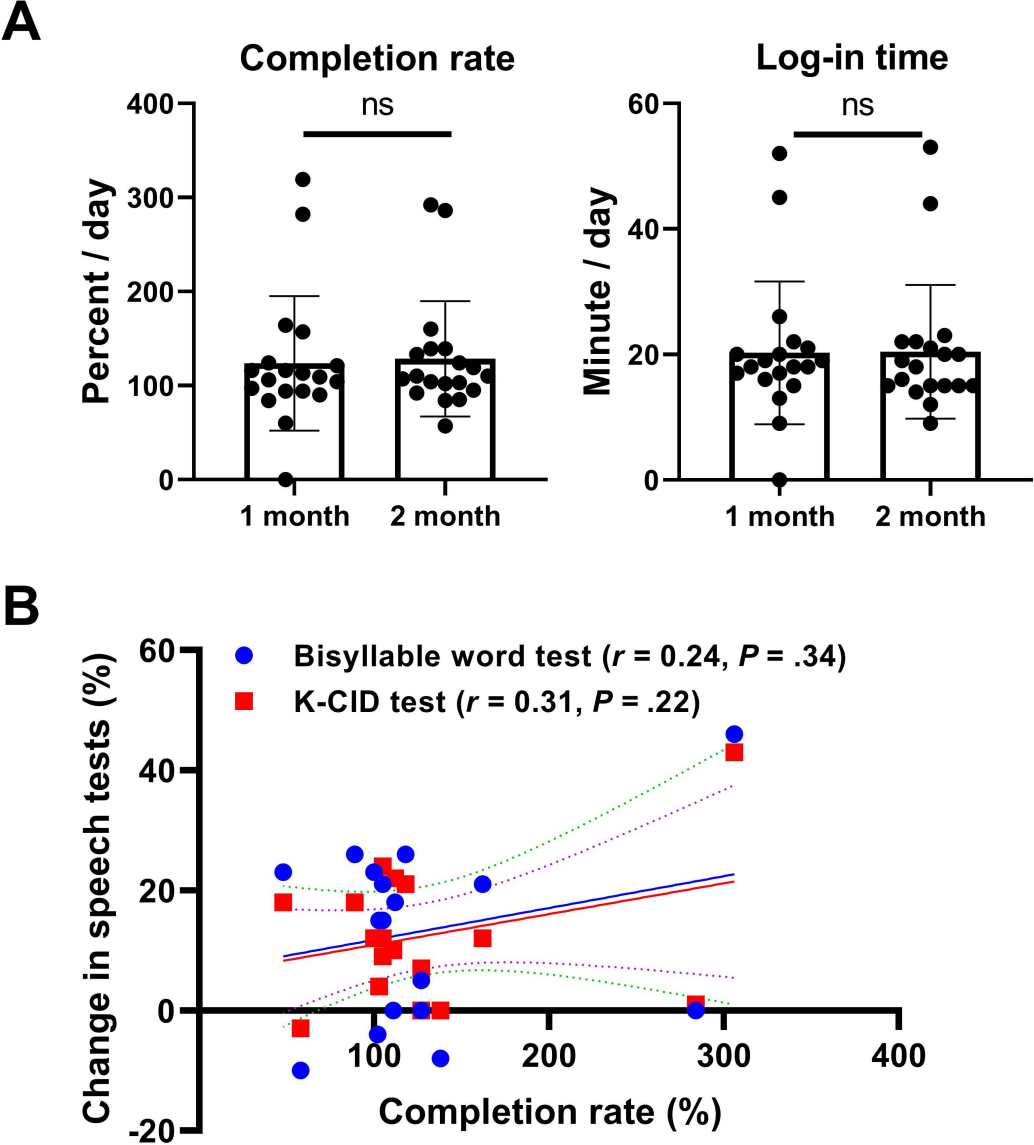
Compliance of chatbot-delivered mobile auditory training program and its correlation with speech perception tests. (A) Completion rate (%) and log-in time (min) were similar in the first month and second month (both *P*>.05; Wilcoxon signed rank test; error bars indicate SD). (B) No significant correlations were observed between compliance and speech perception tests (all *P*>.05, Pearson correlation test). K-CID: Korean version of Central Institute for the Deaf.

Log-in times, completion rates, and scores of all participants were collected by the CMAT program and analyzed. Mean daily application log-in time was 20.3 (SD 11.4) minutes in the first month and 20.4 (SD 10.7) minutes in the second. The completion rate was higher than our recommendation; 123.7% (SD 71.4%) in the first month and 128.5% (SD 61.3%) in the second month. There were no statistically significant differences among these variables (*P*=.80 for completion rate and *P*=.50 for log-in time; Wilcoxon signed rank test) (Figure 5A). The analysis of compliance and speech perception tests revealed no significant correlation (Figure 5B).

## Discussion

### Principal Results

In this study, we developed a new CMAT program in the Korean language, and we confirmed that it was effective in improving speech perception abilities and had high compliance among experienced bilateral HA users.

Our study evaluated speech perception performance at the levels of phoneme, word, and sentence. When the tests were repeated, they all showed improvements in both the ATG and CG. This is a similar finding as our previous report, and it might have been caused by the participants adapting to the test materials [10,30]. Interaction effects implied that compared to the initial time point, at 1 month and 2 months there was a greater performance improvement in the ATG in the bisyllable test and K-CID test. However, no significant findings were observed for the Ling Six Sound test, VCIT, or monosyllable test. The prominent improvement in bisyllable word and sentence perception tests could be explained by the effect of AT having been achieved only to a limited degree for the trained task. The CMAT program used bisyllable words and sentences, which resulted in marked improvement on the tests assessing the same ability. Several previous studies suggested that the effect of AT appeared only in trained tasks and did not lead to improvements in untrained tasks [21,31,32].

All 3 questionnaires measuring subjective hearing disability and HA satisfaction showed no improvement after 2 months of CMAT, a result that is contrary to our previous study. Our previous study investigated the effectiveness of hospital-based AT and showed significant improvements on the K-IOI-HA and K-HHIE questionnaires in the intervention group [10]. A systematic review suggested that telemedicine has similar feasibility as conventional hearing rehabilitation; however, this review paper was not focused on AT; it focused more on HA fitting, device testing, and counseling [33]. To the best of our knowledge, only 3 comparative studies have been conducted of telemedicine and conventional AT [34-36]. A pilot study comparing the feasibility of tablet-based AT and conventional face-to-face AT in adult cochlear implant recipients reported similar results to our study. Even though tablet-based AT showed better speech improvements in tablet-based training, subjective auditory ability measured by the Oldenburger Inventory-R questionnaire showed partial improvement only in the conventional AT group and not in the teletherapeutic group [36]. The reason why we could not find subjective speech perception improvement in the questionnaire seems to be the lack of interaction between the participants and health care providers, who encourage and reassure patients during each session of conventional AT programs. A relatively short study period might be another reason. A long-term follow-up study or a study with participants who are more exposed to health care providers during CMAT might be needed to support our suggestions in the future.

Of the 20 participants who agreed to CMAT, 17 (85%) completed all or more requested training times, and there was no difference in compliance during the 2-month study. Our completion rate is similar to previous studies [10,37], and it seems that mobile-based AT is easily accessible and that the chat-based interface gives users a more interactive impression [8].

In our study, there was no statistically significant correlation between CMAT compliance and listening performance, which might be due to the high and homogeneous completion rate of an average of 120% of the program in the ATG. However, previous studies suggested that training duration is related to the improvement in listening abilities. A study using the Listening and Communication Enhancement training program in veterans using HAs reported that 84% (42/50) of participants completed the required sessions, and improvement in off-task performance was significantly better in the group of participants who completed all the sessions [38]. Another study examined the use of ReadmyQuips among new HA users and reported a correlation between the words-in-noise test and training time but not between the hearing-in-noise test and training time [39].

### Strengths and Limitations

The mobile-based AT program we introduced has several advantages that set it apart from other internet-based AT programs. The first advantage lies in the interface, which features chatting, thus catering to users familiar with mobile messengers and providing a more convenient experience. Simplistic problem-solving might bore users easily, and gamification could come across as juvenile for adult users. Additionally, our interface uses a format in which users chat with a character resembling a medical professional, aiming to give users the impression of receiving remote treatment from experts. The second advantage pertains to the quality and quantity of the provided problems. Other AT programs sometimes present meaningless word lists or scenarios where the sound completely differs from the presented words. However, our CMAT program was designed with the intention of using 1540 frequently used words provided by the National Institute of the Korean Language to offer assistance in users’ everyday lives. We aimed to enhance consonant discrimination abilities that are challenging for individuals with hearing impairments by creating problems that categorize cases where the vowels are similar but the consonants differ. Additionally, due to the vast number of questions, each exceeding 1000, participants might not encounter the same question more than once during the 2-month test period.

Nevertheless, CMAT also has its limitations. Recent artificial intelligence (AI)–powered language models like ChatGPT, which enable real-time interactions, were not integrated. The program did not incorporate various user-responsive technologies such as those found in AI-driven tools. Furthermore, additional settings such as background noise or speech speed adjustments were not possible in this version of CMAT. We refrained from including these adjustable settings to maintain the consistency of the training program and avoid introducing bias in the study. However, in the next version, we plan to incorporate features that allow users to adjust the program to their skill level.

From a clinical study perspective, there are several other advantages. Foremost among them is the fact that this is the first well-designed study conducted using a mobile-based AT program using the Korean language. This study also suggests the possibility that AT could be widely applied to the hearing rehabilitation process by showing the effect of AT on users with HA experience, which has been controversial in previous reports [19-21].

However, several limitations that emerged during the course of this study should also be taken into consideration. First, despite the randomization, the high-frequency hearing threshold in the right ear was significantly higher in the ATG than the CG, indicating that the CG score was better in most initial speech perception tests. We considered a subgroup analysis such as participant matching to avoid differences in initial hearing threshold, but we could not conduct such an analysis due to the limitations of a randomized prospective study with a relatively small number of participants. Instead, we tried to minimize unintentional bias by adjusting the 4 kHz threshold of the PTA when analyzing with the linear mixed model; however, there is a possibility that the effect of CMAT was underestimated due to differences in initial hearing threshold. Further research using a larger number of participants is needed in the future. Second, a 2-month training time and follow-up duration might not be long enough to show the effect of CMAT on all types of hearing performance and subjective satisfaction. Therefore, a future study to evaluate the long-term effects of CMAT with a longer training time is needed. Third, this study was not blinded, and the potential risk of type I error should be considered. Lastly, fitting verification results, such as real-ear measurements, were not included, and data on HA use time during the trial were not included in this analysis. Instead, fitting validation was conducted using sound field tests, and only those who had been using HAs for more than 3 months and for at least 8 hours a day were included in this study. Incorporating more objective fitting verification and HA use time monitoring with data logging would be beneficial in future studies.

Several points for improving this CMAT program can be considered in the future. First and foremost, AI technology can be adapted to enable 2-way interactions between the user and the program. The AI system can generate an almost infinite number of new questions and adjust the question difficulty level based on the user’s responses. Additionally, while this study used a chat-based interface for adults, more casual training methods, such as gamified training, could be adopted for pediatric patients with hearing loss.

### Conclusions

In this study, we developed a novel mobile-accessible AT program with a chat-based interface and showed that it enhanced speech perception ability in experienced HA users. CMAT seems to increase the accessibility of and compliance to AT, and we expect that it will be used in all stages of the hearing rehabilitation process.

## Supplementary material

10.2196/50292 Multimedia Appendix 1 Linear mixed model analysis of speech perception tests after arcsine transformation.

10.2196/50292 Multimedia Appendix 2 Detailed results of 3 questionnaire surveys.

10.2196/50292 Checklist 1 CONSORT eHEALTH checklist (V 1.6.1).

## Abbreviations

AI: artificial intelligence
APHAB: Abbreviated Profile of Hearing Aid Benefit
AT: auditory training
ATG: auditory training group
CG: control group
CMAT: chat-based mobile auditory training
CONSORT: Consolidated Standards of Reporting Trials
HA: hearing aid
K-CID: Korean version of Central Institute for the Deaf
K-HHIE: Korean version of the Hearing Handicap Inventory for the Elderly
K-IOI-HA: Korean version of International Outcome Inventory for Hearing Aids
PTA: pure tone audiogram
VCIT: Vowel and Consonant Imitation Test
