# Supplementary material for: Hearing Rehabilitation With a Chat-Based Mobile Auditory Training Program in Experienced Hearing Aid Users: Prospective Randomized Controlled Study
Source: JMIR Mhealth Uhealth. 2024 Feb 9;12:e50292. doi: 10.2196/50292 (PMC10867308; doi:10.2196/50292)
Supplement: Multimedia Appendix 1 [file mhealth-v12-e50292-s001.docx]

Supplementary 1. Linear mixed model analysis of speech perception tests before and after arcsine transformation.

| **Test** | **Time** | | **Percentage** | | **Arcsine transformation** | |
| --- | --- | --- | --- | --- | --- | --- |
|  |  |  | **ATG** | **CG** | **ATG** | **CG** |
| **Ling-6-sound test** | Initial | Mean  (SD) | 75.44 (17.00) | 80.83 (15.56) | 1.09  (0.23) | 1.18  (0.26) |
|  |  | *P* | 0.343 | | 0.343 | |
|  | 1 M | Mean (SD) | 84.21 (11.75) | 84.17 (13.76) | 1.22  (0.23) | 1.24  (0.26) |
|  |  | *P* | >0.999 | | >0.999 | |
|  | 2 M | Mean (SD) | 87.72  (9.37) | 91.67 (8.55) | 1.26  (0.21) | 1.36  (0.22) |
|  |  | *P* | 0.200 | | 0.132 | |
|  | 1M - Initial | Mean (SD) | 8.77  (16.07) | 3.33 (16.75) | 0.13  (0.22) | 0.06  (0.26) |
|  |  | Within *P* | 0.011* | 0.385 | 0.019* | 0.252 |
|  |  | Between *P* | 0.301 | | 0.441 | |
|  | 2M - Initial | Mean (SD) | 12.28  (17.43) | 10.83  (15.56) | 0.20  (0.25) | 0.18  (0.28) |
|  |  | Within *P* | 0.008* | 0.010* | 0.008* | 0.022* |
|  |  | Between *P* | 0.821 | | 0.938 | |
| **VCIT (Vowel)** | Initial | Mean (SD) | 84.21 (14.60) | 83.93  (17.48) | 1.22 (0.24) | 1.24 (0.29) |
|  |  | *P* | 0.830 | | 0.830 | |
|  | 1 M | Mean (SD) | 91.73  (7.63) | 89.29  (13.61) | 1.33 (0.18) | 1.31 (0.24) |
|  |  | *P* | 0.861 | | 0.861 | |
|  | 2 M | Mean (SD) | 92.48  (9.37) | 94.29  (7.55) | 1.37 (0.22) | 1.41 (0.19) |
|  |  | *P* | 0.656 | | 0.545 | |
|  | 1M - Initial | Mean (SD) | 7.52  (14.18) | 5.36  (12.01) | 0.11 (0.23) | 0.07 (0.24) |
|  |  | Within *P* | 0.033* | 0.051 | 0.044* | 0.330 |
|  |  | Between *P* | 0.615 | | 0.474 | |
|  | 2M - Initial | Mean (SD) | 8.27  (16.54) | 10.36  (15.10) | 0.15 (0.31) | 0.17 (0.27) |
|  |  | Within *P* | 0.043* | 0.005* | 0.048* | 0.011* |
|  |  | Between *P* | 0.943 | | 0.885 | |
| **VCIT (Consonant)** | Initial | Mean (SD) | 58.77  (18.73) | 75.28  (14.02) | 0.90  (0.24) | 1.07  (0.19) |
|  |  | *P* | 0.003* | | 0.003* | |
|  | 1 M | Mean (SD) | 68.71  (18.07) | 77.78  (16.72) | 1.00  (0.23) | 1.11  (0.22) |
|  |  | *P* | 0.112 | | 0.141 | |
|  | 2 M | Mean (SD) | 74.27  (14.61) | 81.67  (18.21) | 1.04  (0.16) | 1.18  (0.26) |
|  |  | *P* | 0.082 | | 0.046* | |
|  | 1M - Initial | Mean (SD) | 9.94  (13.56) | 2.50  (14.02) | 0.11  (0.16) | 0.04  (0.18) |
|  |  | Within *P* | 0.005* | 0.435 | 0.008* | 0.362 |
|  |  | Between *P* | 0.101 | | 0.214 | |
|  | 2M - Initial | Mean (SD) | 15.50  (15.11) | 6.39  (16.25) | 0.18  (0.18) | 0.11  (0.22) |
|  |  | Within *P* | <0.001* | 0.095 | <0.001* | 0.044* |
|  |  | Between *P* | 0.078 | | 0.310 | |
| **Mono-syllable test** | Initial | Mean (SD) | 62.28  (16.71) | 70.87  (11.52) | 0.92  (0.18) | 1.01  (0.13) |
|  |  | *P* | 0.068 | | 0.083 | |
|  | 1 M | Mean (SD) | 70.00  (11.92) | 72.43  (12.61) | 1.00  (0.13) | 1.03  (0.14) |
|  |  | *P* | 0.544 | | 0.521 | |
|  | 2 M | Mean (SD) | 75.89  (11.55) | 81.07  (12.28) | 1.06  (0.14) | 1.14  (0.15) |
|  |  | *P* | 0.062 | | 0.047* | |
|  | 1M - Initial | Mean (SD) | 7.72  (10.78) | 1.57  (10.49) | 0.08  (0.12) | 0.02  (0.12) |
|  |  | Within *P* | 0.006* | 0.512 | 0.014* | 0.472 |
|  |  | Between *P* | 0.067 | | 0.100 | |
|  | 2M - Initial | Mean (SD) | 13.61  (17.60) | 10.20  (10.99) | 0.16  (0.20) | 0.13  (0.13) |
|  |  | Within *P* | 0.003* | <0.001 | 0.006* | <0.001* |
|  |  | Between *P* | 0.476 | | 0.306 | |
| **Bi-syllable test** | Initial | Mean (SD) | 76.14  (18.12) | 84.37  (12.07) | 1.09  (0.23) | 1.21  (0.21) |
|  |  | *P* | 0.102 | | 0.097 | |
|  | 1 month | Mean (SD) | 87.01  (10.07) | 88.39  (9.87) | 1.22  (0.14) | 1.25  (0.15) |
|  |  | *P* | 0.542 | | 0.542 | |
|  | 2 months | Mean (SD) | 88.62  (9.29) | 88.83  (11.13) | 1.25  (0.16) | 1.28  (0.19) |
|  |  | *P* | 0.888 | | 0.665 | |
|  | 1M - Initial | Mean (SD) | 10.87  (12.50) | 4.02  (9.31) | 0.13  (0.16) | 0.04  (0.15) |
|  |  | Within *P* | 0.001* | 0.069 | 0.003* | 0.255 |
|  |  | Between *P* | 0.059 | | 0.087 | |
|  | 2M - Initial | Mean (SD) | 12.48  (14.56) | 4.46  (10.04) | 0.18  (0.20) | 0.07  (0.16) |
|  |  | Within *P* | 0.002* | 0.062 | 0.001* | 0.081 |
|  |  | Between *P* | 0.052 | | 0.063 | |
| **K-CID** | Initial | Mean (SD) | 82.25  (15.53) | 90.15  (9.51) | 1.18  (0.23) | 1.30  (0.19) |
|  |  | *P* | 0.083 | | 0.074 | |
|  | 1 month | Mean (SD) | 91.44  (9.06) | 92.91  (7.73) | 1.31  (0.16) | 1.35  (0.16) |
|  |  | *P* | 0.571 | | 0.480 | |
|  | 2 months | Mean (SD) | 94.03  (7.16) | 96.79  (5.71) | 1.37  (0.16) | 1.46  (0.15) |
|  |  | *P* | 0.116 | | 0.075 | |
|  | 1M - Initial | Mean (SD) | 9.19  (9.46) | 2.76  (6.64) | 0.13  (0.14) | 0.05  (0.13) |
|  |  | Within *P* | <0.001* | 0.095 | <0.001* | 0.123 |
|  |  | Between *P* | 0.017* | | 0.030* | |
|  | 2M - Initial | Mean (SD) | 11.78  (11.13) | 6.64  (7.66) | 0.21  (0.15) | 0.15  (0.16) |
|  |  | Within *P* | <0.001* | <0.001* | <0.001* | <0.001* |
|  |  | Between *P* | 0.108 | | 0.291 | |

ATG, auditory training group; CG, control group; M, month; vs, versus; CIC, complete in the canal; VCIT, Vowel and Consonant Imitation Test; K-CID, Korean version of Central Institute for the Deaf. Within P, Paired t-test or Wilcoxon signed rank test; Between P, Independent t-test or Wilcoxon rank sum test.
