# Supplementary material for: Hearing Rehabilitation With a Chat-Based Mobile Auditory Training Program in Experienced Hearing Aid Users: Prospective Randomized Controlled Study
Source: JMIR Mhealth Uhealth. 2024 Feb 9;12:e50292. doi: 10.2196/50292 (PMC10867308; doi:10.2196/50292)
Supplement: Multimedia Appendix 2 [file mhealth-v12-e50292-s002.docx]

**Supplementary 2.** **Detailed results of three types of questionnaire surveys.**

**A. APHAB (Abbreviated Profile of Hearing Aid Benefit)**

|  |  | **Initial** | **1 month** | **2 months** | **P-value^†^** |
| --- | --- | --- | --- | --- | --- |
| **Total** | ATG | 187.5 (64.8) | 184.7 (54.2) | 189.9 (52.8) | 0.26 |
|  | CG | 169.9 (55.9) | 174.9 (62.6) | 161.5 (60.0) |  |
|  | P-value^††^ | 0.14 | 0.59 | 0.12 |  |
| **Easy of Communication** | ATG | 32.8 (22.1) | 28.6 (24.6) | 28.4 (18.9) | 0.49 |
|  | CG | 24.3 (21.4) | 28.1 (24.3) | 23.6 (22.1) |  |
|  | P-value^††^ | 0.19 | 0.96 | 0.25 |  |
| **Reverberation** | ATG | 48.7 (14.3) | 52.1 (11.9) | 48.8 (17.8) | 0.30 |
|  | CG | 47.5 (14.1) | 48.0 (11.1) | 41.2 (16.6) |  |
|  | P-value^††^ | 0.79 | 0.26 | 0.16 |  |
| **Background noise** | ATG | 44.3 (16.6) | 44.9 (12.4) | 42.6 (19.6) | 0.53 |
|  | CG | 43.8 (13.8) | 45.0 (20.3) | 38.8 (19.9) |  |
|  | P-value^††^ | 0.91 | 0.98 | 0.54 |  |
| **Aversiveness** | ATG | 61.7 (31.0) | 59.2 (28.8) | 70.0 (20.8) | 0.54 |
|  | CG | 54.4 (25.5) | 53.8 (28.7) | 57.9 (26.4) |  |
|  | P-value^††^ | 0.19 | 0.55 | 0.11 |  |

**B. K-HHIE (Korean version of Hearing Handicap Inventory for the Elderly)**

|  |  | **Initial** | **1 month** | **2 months** | **P-value^†^** |
| --- | --- | --- | --- | --- | --- |
| **Total Score** | ATG | 28.4 (26.8) | 31.6 (31.7) | 32.7 (31.8) | 0.81 |
|  | CG | 28.0 (24.3) | 29.1 (26.5) | 34.1 (27.6) |  |
|  | P-value^††^ | >0.99 | 0.97 | 0.82 |  |
| **Emotional Score** | ATG | 12.9 (15.3) | 16.2 (18.6) | 15.8 (18.8) | 0.64 |
|  | CG | 13.0 (12.6) | 13.2 (13.2) | 15.3 (15.5) |  |
|  | P-value^††^ | 0.84 | 0.83 | 0.87 |  |
| **Social/Situational Score** | ATG | 15.5 (12.9) | 15.4 (13.4) | 17.0 (13.7) | 0.81 |
|  | CG | 15.1 (12.9) | 15.9 (13.8) | 18.8 (12.9) |  |
|  | P-value^††^ | 0.91 | > 0.99 | 0.57 |  |

**C. K-IOI-HA (Korean version of International Outcome Inventory for Hearing Aids)**

|  |  | **Initial** | **1 month** | **2 months** | **P-value^†^** |
| --- | --- | --- | --- | --- | --- |
| **Average Score** | ATG | 4.0 (0.4) | 4.1 (0.4) | 4.0 (0.5) | 0.83 |
|  | CG | 3.9 (0.8) | 4.0 (0.7) | 4.0 (0.7) |  |
|  | P-value^††^ | 0.75 | 0.45 | 0.80 |  |
| **Q1** | ATG | 4.6 (0.8) | 4.6 (0.6) | 4.5 (0.7) | 0.13 |
|  | CG | 4.2 (1.2) | 4.57 (0.7) | 4.5 (0.8) |  |
|  | P-value^††^ | 0.35 | 0.78 | > 0.99 |  |
| **Q2** | ATG | 3.9 (1.0) | 4.1 (0.8) | 4.2 (0.9) | 0.85 |
|  | CG | 4.1 (1.2) | 4.2 (0.9) | 4.19 (1.0) |  |
|  | P-value^††^ | 0.31 | 0.55 | 0.80 |  |
| **Q3** | ATG | 3.4 (1.3) | 3.5 (1.0) | 3.4 (1.1) | 0.88 |
|  | CG | 3.5 (1.1) | 3.3 (1.0) | 3.2 (1.0) |  |
|  | P-value^††^ | 0.99 | 0.65 | 0.59 |  |
| **Q4** | ATG | 4.5 (0.6) | 4.2 (0.6) | 4.3 (0.6) | 0.87 |
|  | CG | 4.1 (1.1) | 3.8 (1.0) | 4.0 (1.0) |  |
|  | P-value^††^ | 0.40 | 0.19 | 0.36 |  |
| **Q5** | ATG | 3.3 (1.3) | 4.0 (0.8) | 3.7 (1.1) | 0.37 |
|  | CG | 3.6 (1.2) | 3.8 (1.2) | 3.8 (0.9) |  |
|  | P-value^††^ | 0.51 | 0.79 | 0.81 |  |
| **Q6** | ATG | 4.3 (1.1) | 4.4 (0.9) | 4.2 (1.1) | 0.34 |
|  | CG | 4.0 (1.3) | 4.2 (1.1) | 4.3 (0.9) |  |
|  | P-value^††^ | 0.33 | 0.72 | 0.86 |  |
| **Q7** | ATG | 3.8 (0.8) | 4.0 (0.8) | 3.9 (0.9) | 0.92 |
|  | CG | 3.8 (1.1) | 3.9 (0.9) | 3.8 (1.0) |  |
|  | P-value^††^ | 0.89 | 0.78 | 0.87 |  |

Values are represented as mean (standard deviation). † P values are calculated by linear mixed-effects model. P values are calculated using the Wilcoxon rank sum test or paired t-test for continuous variables. ATG, hearing rehabilitation therapy group; CG, control group.
